# Speech Perception Deficits in Mandarin-Speaking School-Aged Children with Poor Reading Comprehension

**DOI:** 10.3389/fpsyg.2017.02144

**Published:** 2017-12-14

**Authors:** Huei-Mei Liu, Feng-Ming Tsao

**Affiliations:** ^1^Department of Special Education, National Taiwan Normal University, Taipei, Taiwan; ^2^Department of Psychology, National Taiwan University, Taipei, Taiwan

**Keywords:** speech perception, categorical speech perception, lexical tone perception, reading comprehension, Mandarin-speaking children

## Abstract

Previous studies have shown that children learning alphabetic writing systems who have language impairment or dyslexia exhibit speech perception deficits. However, whether such deficits exist in children learning logographic writing systems who have poor reading comprehension remains uncertain. To further explore this issue, the present study examined speech perception deficits in Mandarin-speaking children with poor reading comprehension. Two self-designed tasks, consonant categorical perception task and lexical tone discrimination task were used to compare speech perception performance in children (*n* = 31, age range = 7;4–10;2) with poor reading comprehension and an age-matched typically developing group (*n* = 31, age range = 7;7–9;10). Results showed that the children with poor reading comprehension were less accurate in consonant and lexical tone discrimination tasks and perceived speech contrasts less categorically than the matched group. The correlations between speech perception skills (i.e., consonant and lexical tone discrimination sensitivities and slope of consonant identification curve) and individuals’ oral language and reading comprehension were stronger than the correlations between speech perception ability and word recognition ability. In conclusion, the results revealed that Mandarin-speaking children with poor reading comprehension exhibit less-categorized speech perception, suggesting that imprecise speech perception, especially lexical tone perception, is essential to account for reading learning difficulties in Mandarin-speaking children.

## Introduction

Reading comprehension skills are essential for academic learning and success. Children are required to establish appropriate language comprehension and word decoding skills to become successful and independent readers (Gough and Tunmer, 1986; Cain, 2016). The associations among oral language comprehension, word decoding skills, and reading comprehension have been extensively investigated in typically developing children and children with impaired language and literacy skills when learning the alphabetic writing systems (Cain and Oakhill, 2006; Catts et al., 2006; Oakhill and Cain, 2012; Catts et al., 2015; Lepola et al., 2016).

From a developmental perspective, oral language competency, particularly vocabulary knowledge and listening comprehension, has been found to be concurrently and longitudinally predictive of reading comprehension in children with typical reading skills (Catts et al., 1999; Nation and Snowling, 2004; Lervag et al., 2017). In addition, although a strong correlation exists between word decoding and reading comprehension in beginning readers (Garcia and Cain, 2014; Lervag et al., 2017), in older children, this correlation becomes weaker as the correlation between language and reading comprehension becomes stronger (Diakidoy et al., 2005; Vellutino et al., 2007). This finding suggests that a more dominant language comprehension process contributes to reading comprehension in the later stages of reading development. Clarke et al. (2010) reported that interventions focused on oral language comprehension may have a long-term impact on improving reading comprehension. Such correlational and causal evidence indicates that oral language comprehension during preschool lays the foundation for learning to read (Dickinson et al., 2010) and play an increasingly crucial role in reading development in elementary school children (Storch and Whitehurst, 2002).

With regard to reading disabilities, it has been commonly reported that young children with language impairment, compared with their age-matched peers, exhibit poorer performance in their literacy skill development (Snowling et al., 2000), particularly in reading comprehension (Botting et al., 2006; Bishop et al., 2009). Recent studies showed that some children’s poor reading comprehension may be a consequence of poor language comprehension, in addition to inefficient or inaccurate word decoding skills that limited children’s ability to extract meaning from text (Cain and Oakhill, 2006; Cain, 2016). As in some longitudinal studies, it has been reported that school-aged children who were found to have problems with reading comprehension exhibited deficiencies in their oral language comprehension at younger ages (from toddlers to kindergarten). The result suggests that impaired oral language skills may lead to difficulties comprehending written language (Catts et al., 2006; Nation et al., 2010; Catts et al., 2012; Justice et al., 2013).

In the field of literacy development, the majority of the studies have investigated children’s development of and deficits in word decoding skills, such as dyslexia. Most studies of word-level reading deficits in speakers of alphabetical languages have asserted that phonological processing ability is a vital prerequisite to word reading development (Catts et al., 1999; Vellutino et al., 2004). Although the core processing components underlying phonological deficits in children speaking various languages who have dyslexia are yet to be determined (Chan et al., 2007), an increasing amount of studies are exploring more fundamental process of phonological processing skills, such as speech perception (Blomert and Mitterer, 2004; Veuillet et al., 2007; Vandewalle et al., 2012; Hakvoort et al., 2016). Speech perception deficits may be the fundamental cause of problems in phonological processing and reading (Ziegler et al., 2011; Goswami et al., 2013).

Speech perception involves distinguishing speech sounds and categorizing acoustic signals onto the individual phonemes such as vowels, consonants, and lexical tones to make phonetic identifications and determine meaningful differences between phonetic contrasts. For example, the syllables /ba/ and /da/ can be perceived as two distinct syllables because the initial consonants are categorized into different phonemes. Speech perception deficits have been increasingly observed in children with impaired language and reading abilities (Robertson et al., 2009; Chen and Liu, 2010; Zhang et al., 2012). Numerous studies have employed speech discrimination tasks involving pairs of syllables that differ by only a single phonemic feature. These studies have shown that some children with dyslexia made more phoneme discrimination errors than their age-matched peers without dyslexia (Werker and Tees, 1987; Mody et al., 1997; Adlard and Hazan, 1998; Serniclaes et al., 2001). These findings indicated a weakness in the representations of speech sounds in children with dyslexia.

Categorical perception refers to the process in which listeners extract linguistically relevant acoustic information from a speech continuum to form relatively discrete perceptual categories (e.g., the shift from “ba” to “da”). Acoustic differences between variants that cross phoneme boundaries lead to different phonemic perceptions, whereas acoustic differences between variants within the same phonetic category are frequently overlooked (Liberman et al., 1957). Studies using categorical speech perception tasks have further indicated that children with dyslexia have less well-defined perceptual boundaries among phonetic categories; for example, these children performed relatively poorly compared to their age-matched peers in distinguishing acoustic differences between phonemic categories but better in distinguishing acoustic differences within phonemic categories and their results showed a shallower identification curve (Serniclaes et al., 2001, 2004; Breier et al., 2004; Bogliotti et al., 2008; López-Zamora et al., 2012). Drawing on these studies, the findings indicate that children with dyslexia might respond to phonetically irrelevant acoustic cues and exhibit a weaker speech categorization function than average readers (Werker and Tees, 1987; Serniclaes et al., 2004), and they might perceive speech sounds in the same category as allophones of the same phoneme (Serniclaes et al., 2001).

By conducting a meta-analysis, Noordenbos and Serniclaes (2015) found that children with dyslexia showed reliable deficits in the ability to categorize speech sounds and may process speech differently. Blomert and Mitterer (2004) further argued that speech perception deficits in people with dyslexia are not at the level of rapid auditory transition but rather at that of underlying phonological representation. In addition, previous studies have reported that only school-age dyslexic children with concomitant oral language impairment showed impaired categorical perception (Joanisse et al., 2000; Manis and Keating, 2005; Robertson et al., 2009), thereby reflecting a closer relationship between speech perception deficit and oral language impairment but a weaker relationship between speech perception and word reading difficulty.

### Speech Perception Deficits and Language Comprehension

The link between speech perception and language comprehension has been demonstrated in concurrent and longitudinal studies, which have suggested that fine-grained speech perception abilities are essential to language development. For example, positive relationships have been observed between perceptual abilities at 6 months of age and subsequent expressive vocabulary and syntactic complexity at 24-months of age (Tsao et al., 2004; Kuhl et al., 2005). In addition, studies of preschool and school-age children have shown that speech discrimination skills are related to vocabulary development (Edwards et al., 2002), receptive language (Vance et al., 2009), and reading skills (Wayland et al., 2010). Speech perception that infants develop early in life enables the fine-grained analysis of phonetically relevant speech features. Such perceptual process leads to long-term memory representations of the phonemes necessary for rapid and efficient word segmentation, word recognition, and novel word learning (Jusczyk, 1997; Nittrouer, 2005). Thus, children with a weaker ability to process speech relevant information may have more difficulty establishing higher levels of language representation. Indeed, several studies documented speech perception deficits in children with impaired language skills (Robertson et al., 2009; Ziegler et al., 2005, 2011). Furthermore, speech perception in noise predicted the oral language skills, particularly phonology and comprehension. During development, speech perception deficits likely lead to underspecified phonological representations, which can hinder various aspects of language development.

The aforementioned evidence of less specified phonological representations in children with language impairment has been further supported by studies conducting categorical perception tasks. Studies have shown that children with language impairment perceive speech less categorically than do children with typical language development (Thibodeau and Sussman, 1979; Sussman, 1993; Robertson et al., 2009). For example, the shallower identification curve of a synthetic /ba/-/da/ continuum (Thibodeau and Sussman, 1979), shift locations of perceptual boundaries and less well-defined category boundaries (Sussman, 1993), and inconsistent identification performance along the /ba/-/wa/ continuum (Burlingame et al., 2005). However, some studies have demonstrated that speech perception deficits in children with language impairment may be a consequence of task demands and stimulus properties (Coady et al., 2005, 2007).

As discussed, previous studies mainly focusing on the investigation of children learning alphabetic writing systems have shown that children with language impairments or dyslexia generally exhibit speech perception deficits. However, whether such deficits exist in children who have poor reading comprehension remains uncertain. Since language comprehension is strongly associated with reading comprehension, and that speech perception sensitivities are fundamental to develop language comprehension, it is predicted that speech perception deficits may adversely influence the development of reading comprehension.

### Present Study

To fill the research gap, the objectives of the present study were to explore the extent of speech perception deficits in Mandarin-speaking children with poor reading comprehension and assessed the relationship of the speech perception deficits and reading development. Compared with studies investigating speech perception deficits in children learning alphabetic writing systems, e.g., English, studies of speech perception deficits in Mandarin-speaking children with language impairment or reading difficulties are scant. In Mandarin, vowels and tones serve as compulsory syllable units. Consonants are optional units occurring most commonly at the beginning and occasionally at the end of a syllable. For Mandarin-speaking children, in addition to consonant perception, the ability to discriminate between lexical tones is critical for predicting language performance (Chen and Liu, 2010; Liu et al., 2013). Liu et al. (2013) studied 150 Mandarin-speaking children aged 4–8 years who exhibited typical development (30 children in each age group). The speech perception skills of children were measured with AX phonetic discrimination tasks involving three Mandarin phonetic contrasts (i.e., stops with different articulation places, affricates vs. fricatives, and lexical tone 2 vs. 3). The results revealed that the sensitivity of the discrimination to native speech contrasts improved with age during childhood. In addition, the children’s accuracies to distinguish speech sounds were positively correlated with their vocabulary comprehension scores, and the regression model revealed that lexical tone and stop consonant sensitivity contributed to vocabulary development, and lexical tone sensitivity was one of potent predictors. Although very few studies have focused on speech perception deficits in Mandarin-speaking children with language or reading impairment, one study showed that these children performed less accurately when required to discriminate stop aspirated vs. unaspirated contrast (/ta/-/t^h^a/) and lexical tone pairs (Chen and Liu, 2010). In addition, other studies have documented lexical tone perception deficits in Mandarin-speaking children with dyslexia (Zhang et al., 2012) and in Cantonese-speaking children with language impairment (Wong et al., 2009). These results suggest that speech perception deficits in tonal-language children with language impairment are not only observed at phonetic segment levels (i.e., consonants) but also at the suprasegmental level (i.e., lexical tones).

Therefore, the present study used a broader set of speech stimuli and perception tasks to examine the extent of speech perception deficits in Mandarin-speaking elementary school children with poor reading comprehension. Two pairs of consonants (i.e., affricate /tɕ^h^i/ vs. fricative /ɕ/; bilabial /pa/ vs. alveolar /ta/) and one lexical tone contrast (i.e., tone 2 /i2/ vs. tone 3 /i3/) were utilized to test for the generalization of speech perception deficits in children with poor reading comprehension. From a developmental perspective, these contrasts were acquired with different rates of development, the place contrast was developed earlier than the manner contrast as revealed in studies of articulatory development in Mandarin-speaking children (Zhu and Dodd, 2006; Jeng, 2011). Tones are generally acquired earlier than consonants. Therefore, the inclusion of these consonant and tone contrasts is expected to provide a more comprehensive profile of children’s speech perception deficits. For the speech perception test, phonetic discrimination and categorical perception tasks were used. In the AX discrimination task, listeners were required to distinguish between sound pairs in different categories (/tɕ^h^/vs./ɕ/,/i2/vs./i3/), which only differed in terms of minimal phonemic features. In the categorical perception task, the listeners were required to identify whether each sound belonged to one phoneme category or the other (/pa/ vs. /ta/), and to distinguish between- and within-category sounds, including endpoint and midpoint items in a speech continuum. The results of these tasks provided indications of distinguishing and categorizing speech sounds in Mandarin-speaking children with poor reading comprehension.

With respect to the research samples, the present study recruited elementary school children in Taiwan from second through fourth grade. The participants aged from 8 to 10 years would be suitable for the present study as they would go through a crucial phase of reading development. In Taiwan, most children with reading disabilities are officially screened and clinically diagnosed during this period. Therefore, examining the relationships among speech perception, receptive vocabulary, word recognition, and reading comprehension in this age group is practically and theoretically crucial.

The present study examined whether the speech perception deficits reported in children who have language impairment or dyslexia extend to children with reading comprehension difficulties. Furthermore, exploring speech perception in Mandarin-speaking children with reading comprehension difficulties would assess perceptual roles of both phonetic segmental and suprasegmental units in the reading development of children learning logographic writing systems.

## Materials and Methods

### Participants

This study enrolled Mandarin-speaking children with poor reading comprehension and age-matched typically developing peers studying in the grades 2–4 of elementary schools in Taipei City and New Taipei City, Taiwan. A total of 38 children with “reading difficulty” were initially referred by their classroom teachers based on diagnoses made by the Special Education Divisions of the Education Departments in the Taipei City and New Taipei City Governments. A series of standardized language, reading, and cognitive tests were administered to determine the children’s eligibility for participation. To be eligible for participation, a child was required to achieve a standard score above 85 on the Test of Non-verbal Intelligence, Third Edition (Wu et al., 2006). After screening, 31 children who had scored below the 25th percentile rank on the standardized reading comprehension test (Ko and Chan, 2007) were classified as having poor reading comprehension (mean age: 8; 6, 19 boys, age range = 7;4–10;2) (reading difficulty group, RD group). Another 31 age-matched children (mean age: 8;7, 18 boys, age range = 7;7–9;10) who scored above the 25th percentile rank on the reading comprehension and word recognition tests were recruited from the same classes (chronological age matched group, CA group). A total of 62 children participated in this study. According to parental reports and developmental records, none of these children had neurological disorders, pervasive developmental deficits, or significant sensory impairments. This study was carried out in accordance with the recommendations of ‘The Research Ethics Committee of National Taiwan University’ with written informed consent from all subjects and their parents. All subjects gave written informed consent in accordance with the Declaration of Helsinki. The protocol was approved by the “Research Ethics Committee of National Taiwan University.”

## Experimental Materials and Procedures

### Standardized Tests

The Reading Comprehension Screening Test for Elementary School Students (Grades 2–6) (Ko and Chan, 2007), which is the subtest of the Diagnostic Procedure and Test for Mandarin Children with Reading Disabilities, was used to measure Taiwanese school-aged children’s reading comprehension ability at the text level. The internal reliability of the test is 0.70–0.86, and the test–retest reliability is 0.70–0.94. The form A of the test was adopted and would be individually administered to all participants. The reading passages of the test were drawn from grade-appropriate sources that children might encounter in school. During the test, children were asked to read each passage silently, and then were required to mark the correct answers of multiple-choice questions corresponding to the passage. There were 19, 27, and 32 items for the 2nd, 3rd, and 4th graders, respectively. The raw and percentile scores would be calculated, which served as indices for the children’s reading comprehension ability. The participants were defined as having poor reading comprehension if their scores in reading comprehension test were below the 25th percentile according to the cutoff point established by this standardized test.

The Graded Chinese Character Recognition Test (Huang, 2001), another subtest of the Diagnostic Procedure and Test for Mandarin Children with Reading Disabilities, was used to assess the children’s word recognition ability at the character level. The participants were assessed individually, and they read aloud a series of 200 words in decreasing order of printed word frequency and stopped after 20 consecutive errors had been made. The test–retest reliability was 0.81– 0.95 with an interval of 4–6 weeks. The internal reliability and split-half reliability were both 0.99. The accuracy of the children’s responses, including their raw scores and percentiles, was used to index their word recognition ability.

The Peabody Picture Vocabulary Test-Revised (PPVT-R; Lu and Liu, 1998) was used to measure receptive vocabulary performance. In each trial, the child was shown four numbered pictures on a page and asked to point to or to say the number of the picture corresponding to the word spoken by the administrator. The split-half reliability was 0.90–0.97, and the test–retest reliability was 0.90. The raw scores and percentiles were used as oral vocabulary comprehension measures.

### Speech Perception Tasks

#### Speech Discrimination Tasks

For the speech discrimination task, two pairs of Mandarin speech stimuli were used, namely the affricate vs. fricative contrast (i.e., /tɕ^h^i/ vs. /ɕi/) and tones 2 vs. 3 (i.e., /i2/–/i3/) to assess individuals’ speech discrimination sensitivities at both consonantal and suprasegmental levels. The speech stimuli were computer synthesized at a sampling rate of 22,050 Hz and 16-bit resolution. In addition, the sound stimuli were normalized to root mean square amplitude by using Sound Forge 9.0. In the initial consonant contrast, a minimal pair of affricate /tɕ^h^i/ and fricative /ɕi/ was contrasted with the same articulation place but in different manners by manipulating the frication duration and amplitude rising time. The durations of both syllables were 275 ms, and the frication durations of the affricate /tɕ^h^/ and fricative /ɕ/ were 100 and 130 ms, respectively. The maximum amplitude of frication noise occurred at 30 and 100 ms, respectively, and the vowel lengths were 175 and 145 ms, respectively. The vowel formant frequencies were set at 293, 2274, 3186, and 3755 Hz, and the bandwidths were at 80, 90, 150, and 350 Hz, respectively.

For the tonal contrast, a pair of tones 2 and 3 (/i2/ – /i3/) was contrasted. This tonal contrast was acquired later than other tone contrasts (Wong et al., 2005; Zhu and Dodd, 2006), and discrimination of this sound pair is predictive of language development in Mandarin-speaking children (Chen and Liu, 2010). Tone 2 was synthesized with the pitch curve starting point at 219 Hz, lowest point at 195 Hz (with the turning point occurring after 34% of the total duration had elapsed), and a peak of 245 Hz at the end. Pitch of Tone 3 was synthesized with the starting point at 216 Hz, lowest point at 156 Hz (with the turning point occurring after 71% of the total duration had elapsed), and end point at 209 Hz. The acoustic parameters of the vowels were F1 = 290 Hz (bandwidth = 100 Hz), F2 = 2815 Hz (bandwidth = 220Hz), F3 = 3945 Hz (bandwidth = 115 Hz), and F4 = 4973 Hz (bandwidth = 239 Hz). The vowel duration was 270 ms.

##### Procedures

The AX discrimination task was used to assess the children’s consonant and tone discrimination accuracies. The instruction for children was “please listen to the sound pairs and then judge whether these sound pairs are same or different.” Each child consecutively received two speech discrimination blocks in randomly assigned sequences (i.e., consonant discrimination or tone discrimination first). After listening to a pair of sounds, the child was required to judge whether the two sounds were the same or different and then responded quickly by pressing the corresponding key on the keyboard. Each sound pair generated four different combinations (AA, AB, BA, BB), and each combination occurred four times. Therefore, 32 trials were conducted (two sound pairs × four combinations × four repetitions). The sound stimulus sequence in each block was arranged randomly. The accuracy rate (%) was computed by dividing the number of accurate responses by the total number of trials. A *d*′ value for each discrimination task served as the measure of each child’s discrimination sensitivity.

Before testing, four practice trials were conducted to help familiarize the children with the experimental procedures. In addition, to reduce cognitive processing and memory loading during the discrimination task, cartoon characters were used as visual aids and were displayed alongside the sound stimuli on the notebook screen. For example, two SpongeBobs indicated that the two sounds were identical, whereas one SpongeBob and one Patrick indicated that the two sounds were different. Feedback for the correct judgment was provided in the practice trials and no feedbacks in the testing trials.

#### Categorical Speech Perception Task

The contrast between the unaspirated stops /pa4/ and /ta4/ in Mandarin was used to create a continuum of eight sounds that were identical except for the second formant transition frequencies between 250 and 2000 Hz with an interval of 250 Hz between two stimuli. The stimuli were synthesized using Praat software recorded at a sampling rate of 44.1 kHz. The duration of each stimulus was 260 ms. This contrast with two consonants of which have different articulation places, is a crucial phonetic contrast in Mandarin (Chen and Liu, 2010). This task was used to assess children’s abilities to categorize speech sounds along an acoustic continuum.

##### Procedures

The identification and AX discrimination tasks were used to assess the children’s categorical speech perception in a series of eight unaspirated stops along the articulation place continuum (/p/–/t/). The instruction for children was “please listen to the sound and then judge whether the sound is /pa4/ or /ta4/”. In the identification task, after listening to one of the eight synthesized sounds in each trial, each child was required to immediately judge whether the sound was /pa4/ or /ta4/ and then responded by pressing the corresponding key on the keyboard. Simultaneously, two traditional Chinese characters that, respectively, corresponded to /pa4/ and /ta4/ and denoted the Chinese meanings of “father” and “big” were displayed on the screen. Eight practice trials were conducted before testing, and feedback was provided after each practice trial. The testing phase consisted of 64 trials (8 sound stimuli × 8 repetitions). The sequence of stimuli was presented in a random order, and no feedback was provided to the children in testing trials. An identification percentage for each sound was calculated based on the child’s responses (e.g., /pa4/ = XX%, /ta4/ = XX%). The results of the identification percentage formed an identification curve, and the slope of each curve was quantified by fitting to a logistic function, where a β (slope coefficient) parameter was generated to index each child’s sensitivity to categorical speech perception. A higher β-value indicated a shallower curve, which was interpreted as relatively weak categorization. Each β parameter was used in the statistical tests to compare the phonetic identification performance of the two groups.

In the AX discrimination task, eight practice trials were conducted before testing, and feedback was provided after each practice trial. Testing consisted of 84 trials including 56 different-by-1 pairs (i.e., the pairs of 1–2, 2–3, 3–4, 4–5, 5–6, 6–7, and 7–8 were repeated eight times), 16 identical pairs (i.e., the pairs of 1–1, 2–2, 3–3… 8–8 each appeared twice), and 8 different-by-7 pairs (the 1–8 pair). To reduce memory loading during the discrimination task, cartoon characters were displayed alongside the sound stimuli on the screen to indicate that the two sounds were identical (i.e., two SpongeBobs) or the two sounds were different (i.e., Spongebob and Patrick). The presentation order of each trial was random for each participant. The proportion of discriminations made for the seven different-by-1 pairs indicated a child’s categorical perception through comparison of between- and within-category discrimination. The “same” pairs and different-by-7 pairs were included in the task to provide the clearest possible cases of each response type and help maintain each child’s focus during the task. The boundaries between phoneme categories for each group were determined based on a visual inspection of peak discrimination.

The children were tested individually in a quiet room. The standardized tests were administered in two 40-min sessions at an interval of no more than 2 weeks. The speech perception tasks were conducted on a separate day by using a notebook (IBM X60) with the psychology software E-Prime (Psychology Software Tools, version 2.0) to present speech stimuli and record child’s responses. The sequences of discrimination tasks (i.e., affricate vs. fricative consonant and lexical tone discrimination) and categorical speech perception task were counterbalanced between participants. During the experiment, each participant wore headphones (Sennheiser HD438) and listened to the sound stimuli played through the notebook with the volume adjusted to a comfortable and clear level (sound pressure level of approximately 68 dB).

### Data Analysis

This study examined speech perception in children with and those without poor reading comprehension and its relationships with receptive vocabulary, word recognition, and reading comprehension. Regarding speech discrimination, a 2 × 2 mixed analysis of variance (ANOVA) was conducted to examine the effects of group (between-subject factor: CA vs. RD) and speech (within-subject factor: consonant vs. tone) contrasts on speech discrimination sensitivity (including the percent correct and *d*′). Regarding categorical speech perception, the proportion of /ta/ identification responses in each of the eight speech stimuli steps was used as the dependent variable, and a mixed ANOVA was conducted. Separate one-way ANOVAs were conducted to examine the group differences in each speech perception measures, including the average proportion of discriminations made for the different-by-7 pairs, within-category pairs and between-category pairs, and β value of the identification curves, between the two groups. In addition, Pearson correlation analysis was conducted to assess the association of speech perception with vocabulary, word recognition, and reading comprehension. Partial correlation analyses were also conducted to examine the correlations while controlling for age as this study included the participants with a wide age range. Finally, we performed hierarchical regression analyses to examine the relative contributions of speech perception measures to reading comprehension while controlling for receptive vocabulary and word recognition.

## Results

The language comprehension, word recognition, reading comprehension, and non-verbal IQ of the participants were assessed using the standardized tests (**Table 1**). The results showed that age and non-verbal IQ did not differ significantly between the RD and CA groups [*t*_(60)_ = 0.357, *p* = 0.722 and *t*_(60)_ = 1.272, *p* = 0.208, respectively]. The RD group performed significantly lower in reading comprehension, word recognition, and receptive vocabulary than did the CA group [*t*_(60)_ = 12.485, *t*_(60)_ = 4.951, *t*_(60)_ = 5.180, respectively, *p* < 0.001 for all tests].

**Table 1:** Group comparisons of age, non-verbal IQ, reading comprehension, word recognition, and language comprehension.

Measures	CA group (*n* = 31)				RD group (*n* = 31)				*t*_(60)_
	*M*	*SD*	Minimum	Maximum	*M*	*SD*	Minimum	Maximum	
Age (month)	103.1	8.1	91	118	102.3	9.6	88	122	0.722
Non-verbal IQ	104.1	7.6	88	120	101.5	8.6	86	119	1.272
Reading comprehension									
Raw score	17.5	3.1	13	24	7.6	3.1	2	12	12.485^∗∗∗^
Percentile	74.8	16.7	43	93	11.3	8.2	1	25	
Chinese character recognition									
Raw score	78.3	22.9	38	120	50.3	21.6	12	97	4.951^∗∗∗^
Percentile	79.8	19.1	35	99	38.6	29.9	1	94	
Receptive vocabulary (PPVT)									
Raw score	104.0	11.8	78	123	81.2	21.4	42	118	5.180^∗∗∗^
Percentile	89.4	14.2	47	99	58.4	32.6	7	94	

*The statistical analyses were performed only on the raw scores. ^∗^*p* < 0.05, ^∗∗^*p* < 0.01, ^∗∗∗^*p* < 0.001.*

### Speech Discrimination in Both Groups

**FIGURE 1:**
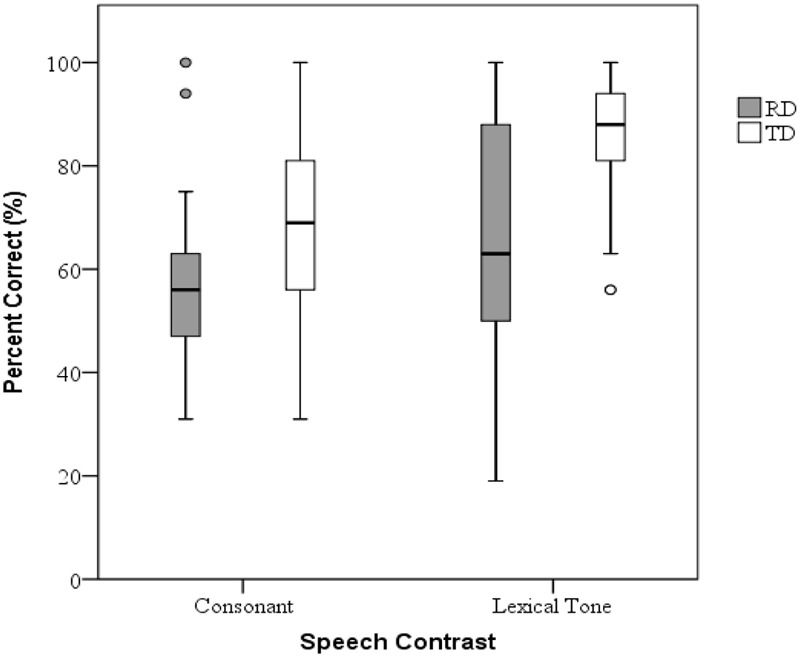
Percent corrects of speech discrimination in two groups.

The group performance of speech discrimination measures (i.e., consonants and tones) are shown in **Table 2** and **Figure 1**. To compare the discrimination task performance of both groups, a mixed ANOVA was conducted on percent corrects with group (CA vs. RD) as the between-subject factor and speech contrasts (consonant vs. tone) as the within-subject factor. The main effects of group [*F*(1,60) = 15.442, *p* < 0.001, η^2^ = 0.205] and speech contrasts [*F*(1,60) = 33.779, *p* < 0.001, η^2^ = 0.360] were significant. The interaction effect of group and speech contrasts did not reach significance [*F*(1,60) = 2.493, *p* = 0.120, η^2^ = 0.040]. We found similar results for the measure of *d*′, the index for speech perception sensitivity, with significant effects of group [*F*(1,60) = 14.456, *p* < 0.001, η^2^= 0.194] and speech contrasts [*F*(1,60) = 35.574, *p* < 0.001, η^2^ = 0.372] but non-significant interaction [*F*(1,60) = 2.289, *p* = 0.120, η^2^ = 0.037]. These results indicated that the RD group performed more poorly compared to the CA group in discerning both the affricate vs. fricative contrast and tone 2 vs. 3 contrast. In addition, the children exhibited lower sensitivity to the affricate–fricative contrast than to the tonal contrast. This finding indicated that children at this age have varying levels of sensitivity to different speech contrasts.

**Table 2:** Means and standard deviations for speech perception measures by group.

Measures	RD (*n* = 31)			CA (*n* = 31)		
	*M* (*SD*)	Maximum	Minimum	*M* (*SD*)	Maximum	Minimum
Consonant discrimination						
Percent correct (%)	0.57 (0.17)	1.00	0.31	0.68 (0.17)	1.0	0.31
*d*′	0.45 (0.99)	3.07	-1.22	1.06 (0.99)	3.07	-0.99
Tone discrimination						
Percent correct (%)	0.68 (0.22)	1.00	0.19	0.86 (0.13)	1.00	0.56
*d*′	1.10 (1.29)	3.07	-1.82	2.16 (0.84)	3.07	0.32
Identification slope (β)	0.39 (0.28)	1.09	0.08	0.24 (0.15)	0.67	0.08

### Categorical Perception in Two Groups

**FIGURE 2:**
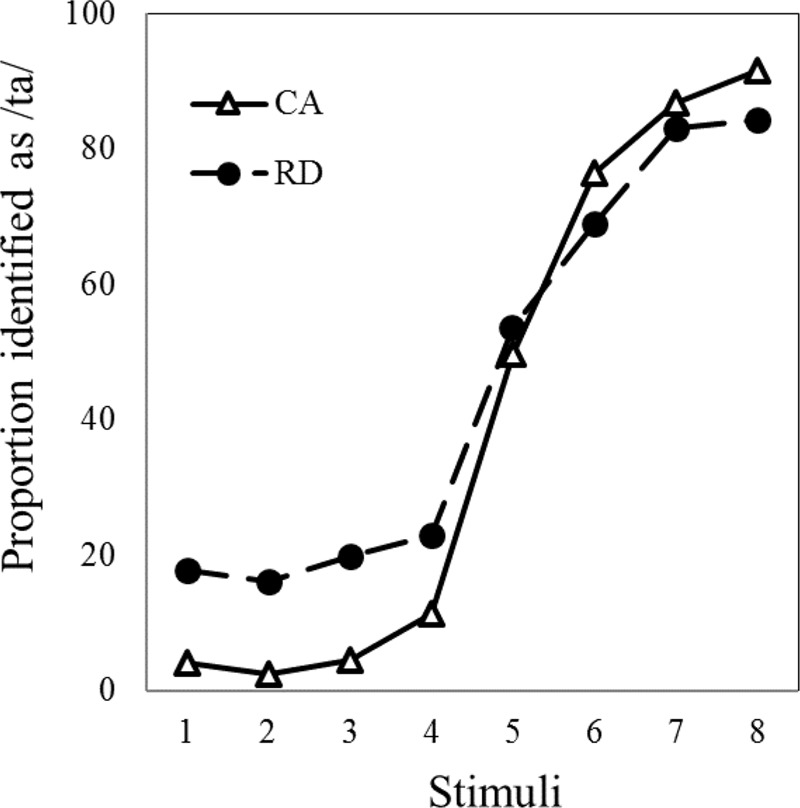
Speech categorization curves in two groups.

**Figure 2** presents the group identification curves for the stop consonants in both groups. The slopes of the identification curves are presented in **Table 3**. Extents of categorizing speech sounds are reflected by the slope coefficients (β) of the identification curve; higher β-values indicate shallower curves and poorer categorical perception. The results of the planned comparison with an independent *t*-test showed that the average slope was significantly higher in the RD group (*M* = 0.39; *SD* = 0.28) than in the CA group (*M* = 0.24; *SD* = 0.15), with *t*_(60)_ = 2.699 and *p* = 0.01. Thus, when identifying speech sounds, the children with poor reading comprehension exhibited less categorized perception than those with typical development.

**Table 3:** Group means (and *SD*) for the proportion of discrimination.

Speech pairs/proportion discriminated (%)	CA group (*n* = 31)		RD group (*n* = 31)	
	*M*	*SD*	*M*	*SD*
Different-by-7 pairs (1–8)	0.86	0.17	0.73	0.20
Between-category (4–5)	0.59	0.19	0.44	0.22
/pa/ within-category (1–2, 2–3, 3–4)	0.12	0.11	0.21	0.19
/ta/ within-category (5–6, 6–7, 7–8)	0.40	0.11	0.38	0.13
Same pairs	0.15	0.12	0.19	0.16

In addition to the identification slope, the proportion of /ta/ responses in each of the eight speech stimulus steps was used as the dependent variable, and a mixed ANOVA was conducted. We found significant main effects for group [*F*(1,60) = 12.90, *p* = 0.001, η^2^ = 0.177] and speech stimuli [*F*(7,420) = 203.499, *p* < 0.001, η^2^= 0.772] and a significant interaction effect of group and sound stimuli [*F*(7,420) = 2.603, *p* = 0.012, η^2^ = 0.042]. Additional analyses of the simple main effects revealed that the CA [*F*(7,240) = 134.116, *p* < 0.001, η^2^ = 0.796] and RD [*F*(7,240) = 58.620, *p* < 0.001, η^2^ = 0.631] groups responded differently to the eight stimulus steps. Bonferroni *post hoc* comparisons revealed that both groups had a higher /ta/ identification rate in stimuli 5, 6, 7, and 8 than in stimuli 1, 2, 3, and 4 (*p*_s_ < 0.01), indicating that the categorization boundary of the two groups fell between the fourth and fifth sound stimuli in the /pa/–/ta/ continuum. Among the eight sounds, the RD group was more likely than the CA group to identify stimuli 1, 2, 3, and 4 with /ta/ (*p*_s_ < 0.01), whereas no group differences were found for the other sound stimuli (*p*_s_ > 0.05). These findings revealed the same pattern as that in the slope analyses, suggesting that the RD group had poorer categorical speech perception than did the CA group, particularly with a higher proportion of /ta/ responses to /pa/ stimuli.

**FIGURE 3:**
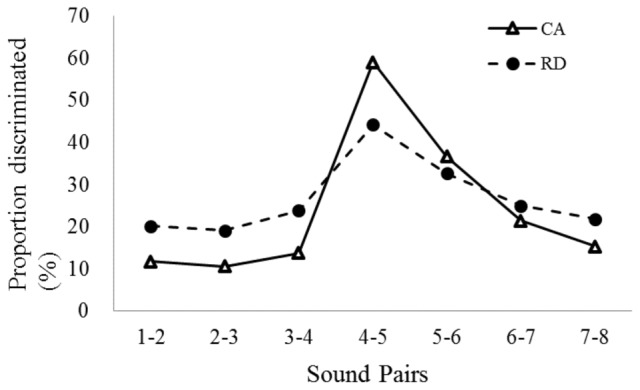
Speech discrimination curves for different-by-1 pairs in two groups.

The discrimination curves of the seven different-by-1 pairs for each group are presented in **Figure 3**. First, we examined the discrimination performance of the endpoint stimuli with different-by-7 pairs in both groups (sound pairs 1–8). The discrimination proportions in the CA (*M* = 0.85; *SD* = 0.17) and RD (*M* = 0.73; *SD* = 0.20) groups were above the chance level (*p* < 0.05). However, the RD group was significantly poorer than the CA group in distinguishing endpoint stimuli [*t*_(60)_ = 2.359, *p* < 0.05]. Subsequently, a mixed ANOVA (between subject factor = group, within subject factor = sound pairs) was conducted to compare the discrimination accuracies of the seven different-by-1 pairs along the continuum in both groups. The results revealed a significant main effect of sound pairs [*F*(6,360) = 27.736, *p* < 0.001, η^2^ = 0.316] and a significant interaction between sound pair and group [*F*(6,360) = 2.946, *p* = 0.008, η^2^ = 0.047] but no significant effect of group [*F*(1,60) = 1.732, *p* = 0.193, η^2^ = 0.028]. Because the sound pair effect was significant, the results of Bonferroni *post hoc* tests (*p* < 0.05) revealed that pair 4–5 had a significantly higher discrimination proportion than did the other pairs (within-category /pa/ pairs: 1–2, 2–3, and 3–4; within-category /ta/: 5–6, 6–7, and 7–8), which suggest that pairs 4–5 are the categorical speech perception boundary for /p/ vs. /t/ contrast. **Table 3** displays the discrimination of different sound pairs in both groups. When combining the performance of the identification curves with the discrimination proportion of between- and within-category sounds, both groups showed a lower discrimination proportion for the within-category sound pairs and a higher proportion of discrimination for the between-category sound pair.

Because the interaction of group and sound pairs suggested the possible occurrence of a group effect among the various different-by-1 pairs, we conducted 7 separate ANOVAs to compare the group differences of discrimination proportion of each pairs, including the within-category conditions (i.e., pairs 1–2, 2–3, 3–4 for /pa/, pairs 5–6, 6–7, 7–8 for /ta/) and between-category condition (pairs 4–5). The results revealed that the two groups had significantly different discrimination proportions for the between- category pair [4–5; *F*(1,60) = 5.907, *p* = 0.018, η^2^ = 0.09]. The CA group had a higher discrimination proportion than did the RD group for the between-category discrimination. The RD group had higher discrimination proportion than did the CA group in the within-category condition for /pa/, [*F*(1,60) = 5.121, *p* = 0.027, η^2^ = 0.079, but not for the /ta/, *F*(1,60) = 0.456, *p* > 0.05]. These findings indicated that compared with the children with typical development, RD group tended to attend to the acoustic differences among the within-category pairs, particularly those in the /pa/ category, but are less accurate to distinguish the same acoustic difference between phonetic categories. In other words, the children with reading difficulties had less specified phonological representations, less abrupt boundaries of phonetic categories, and not well-classified phonetic representations than did the CA group.

### Correlating Speech Perception with Language and Reading Measures

**Table 4** (above diagonal) shows that speech perception measures were generally correlated with vocabulary and reading comprehension in school-aged children. Regarding speech perception of different sound contrasts, lexical tone discrimination was moderately correlated with the receptive vocabulary (*r* = 0.56, *p* < 0.01) and reading comprehension (*r* = 0.58, *p* < 0.01) and was fairly correlated with word recognition (*r* = 0.37, *p* < 0.01). By contrast, consonant discrimination showed fair correlation with the receptive vocabulary (*r* = 0.40, *p* < 0.01), reading comprehension (*r* = 0.40, *p* < 0.01), and word recognition (*r* = 0.27, *p* < 0.05). These findings showed that the children with higher sensitivity to distinguish phonetic contrasts generally perform better in receptive vocabulary, word recognition, and reading comprehension.

**Table 4:** Correlations among speech perception, vocabulary comprehension, word recognition, and reading comprehension, with (below diagonal) and without (above diagonal) controlling for age.

		1	2	3	4	5	6	7	8
1	PPVT	1	0.64**	0.51**	-0.32*	0.56*	0.40**	-0.25*	-0.02
2	Reading comprehension	0.63**	1	0.62**	-0.39**	0.58**	0.40**	-0.29*	-0.08
3	Word recognition	0.49**	0.61**	1	-0.20	0.37**	0.27*	0.026	0.01
4	Identification slope (β)	-0.27*	-0.37**	-0.12	1	-0.43**	-0.30*	-0.45**	0.16
5	Tone discrimination *d*′	0.56**	0.58**	0.38**	-0.46**	1	0.46**	-0.45**	-0.35**
6	Consonant discrimination *d*′	0.36**	0.38**	0.21	-0.26*	0.46**	1	-0.21	-0.14
7	/pa/ within category	-0.26*	-0.30*	0.02	0.48**	-0.45**	-0.22	1	0.12
8	/ta/ within category	-0.01	-0.05	0.02	0.16	-0.35**	-0.14	0.12	1

*1 = PPVT; 2 = reading comprehension; 3 = word recognition; 4 = identification slope (β); 5 = tone discrimination d’; 6 = consonant discrimination d′; 7 = /pa/ within category; 8 = /ta/ within category. Zero-order correlations and partial correlations controlling for age were reported. ^∗^*p* < 0.05, ^∗∗^*p* < 0.01, ^∗∗∗^*p* < 0.001.*

Regarding categorical speech perception, the identification slope (β-value) was negatively correlated with reading comprehension (*r* = -0.39, *p* < 0.01) and receptive vocabulary (*r* = -0.32, *p* < 0.05). This finding indicated that the shallower the identification curve, the poorer is the performance in receptive vocabulary and reading comprehension. However, the identification slope was not significantly associated with word recognition (*p* > 0.05). In addition, the discrimination proportion of /pa/ for the within-category sound pairs was negatively associated with receptive vocabulary and reading comprehension (*r* = -0.25, *p* < 0.05; *r* = -0.29, *p* < 0.05), but the discrimination proportion of /ta/ for the within-category sound pairs did not show any association. The children with higher discrimination proportions for within-category sound pairs may be sensitive to the phonetically irrelevant acoustic changes and thus had poorer performance in receptive vocabulary and reading comprehension.

In addition, results of the partial correlation analyses controlling for age showed that differences between the two types of correlational analyses were very minor (**Table 4**, below diagonal). However, after controlling for age, consonant discrimination sensitivity was not correlated with word recognition (*r*_p_ = 0.21, *p* > 0.05).

Overall, the relationships of speech perception measures with vocabulary and reading comprehension were stronger than that between speech perception measures and word recognition. Compared with measures of consonant perception, the lexical tone discrimination sensitivity of Mandarin-speaking children participating in the current study was more consistently correlated with their vocabulary comprehension, word recognition, and reading comprehension abilities.

### Hierarchical Multiple Regression

Based on correlation analyses, hierarchical multiple regression analysis was conducted to determine the unique predictive power of lexical tone discrimination for reading comprehension while controlling for other related factors. In the first regression model, the children’s reading comprehension scores were set as the dependent measure, and their chronological ages were entered into the model in Step 1 followed by their non-verbal IQ, receptive vocabulary performance, word recognition, and tone discrimination sensitivity, which were independent variables in subsequent steps. The results (**Table 5**) revealed that lexical tone discrimination sensitivity was a significant predictor of reading comprehension and accounted for 5.2% of the variance [Δ*R*^2^ = 0.052, *F*(1,56) = 6.784, *p* < 0.05] after age, non-verbal IQ, receptive vocabulary performance, and word recognition had all been entered. Together, the variables accounted for 59.6% of the variance in reading comprehension [*R* = 0.772, *R*^2^ = 0.596, *F*(5,56) = 16.545, *p* < 0.001]. In two other hierarchical regression models, lexical tone discrimination sensitivity was a significant predictor of vocabulary comprehension and word recognition in the regression models [Δ*R*^2^ = 0.218, *F*(1,58) = 20.851, *p* < 0.001 and Δ*R*^2^ = 0.078, *F*(1,58) = 5.724, *p* < 0.05, respectively] after age and non-verbal IQ had been entered into the models [*R*^2^ = 0.395, *F*(3,58) = 12.598, *p* < 0.001 and *R*^2^ = 0.213, *F*(3,58) = 5.231, *p* < 0.01, respectively].

**Table 5:** Hierarchical regression models of reading comprehension.

Step	Independent variables	Dependent variable				
		Reading comprehension (raw)				
		Final β	*p*	Total *R*^2^	Δ*R*^2^	*p*
1	Age (months)	-0.017	0.855	0.092	0.092	^∗^
2	Non-verbal IQ (raw)	0.130	0.150	0.093	0.001	
3	Receptive vocabulary (raw)	0.346	0.004	0.443	0.350	^∗∗∗^
4	Word recognition (raw)	0.361	0.001	0.546	0.103	^∗∗∗^
5	Tone discrimination (d′)	0.272	0.011	0.596	0.050	^∗^

*^∗^*p* < 0.05, ^∗∗^*p* < 0.01, ^∗∗∗^*p* < 0.001.*

In summary, the results of regression analyses indicated that lexical tone discrimination accuracy is a positive predictor of receptive vocabulary, word recognition, and reading comprehension for Mandarin-speaking children. This finding suggested the importance of lexical tone perception in the development of language and reading comprehension and word recognition.

## Discussion

As a complex cognitive process, reading comprehension is assumed to be a product of word decoding and language comprehension (Gough and Tunmer, 1986). Although many studies demonstrated speech perception deficits in individuals with language impairment or dyslexia (Serniclaes et al., 2004; Ziegler et al., 2005; Robertson et al., 2009), no empirical evidence has been reported regarding the role of speech perception deficits in poor reading comprehension. In the present study, Mandarin-speaking children with poor reading comprehension performed less accurately in consonant and lexical tone discrimination and exhibited poorer speech categorical perception than those in the control group. Notably, speech perception abilities were correlated with their language and reading comprehension performance. These results provide evidence that children with poor text reading comprehension exhibit lower abilities to distinguish phonetic contrasts and less categorized perception for speech sounds. Previous studies have provided evidence of speech perception deficits in some children with oral language or word reading deficiencies (Robertson et al., 2009; Chen and Liu, 2010; Zhang et al., 2012). The results of the present study further support that deficits in fundamental speech perception may influence oral language processing and text reading.

The results of the AX speech discrimination tests revealed that the children with poor reading comprehension showed poorer phonetic discrimination for segmental consonant (/tɕ^h^i/ vs. /ɕi/) and suprasegmental tone (/i2/ vs. /i3/) contrasts than did the control group. This may reflect the inferior ability of the children in the RD group to phonetically distinguish between meaningful differences among speech contrasts. The two phonetic contrasts target the subtle acoustic cues, e.g., duration of frication time in consonant contrast and fundamental frequency contour in tone contrast. The present study employed the AX discrimination task, which greatly reduced cognitive loading. The results are consistent with the findings that children with language impairment or dyslexia exhibit speech processing deficits (Werker and Tees, 1987; Serniclaes et al., 2004; Ziegler et al., 2005; Robertson et al., 2009). Deficits in detecting fine-grain acoustic cues of phonetic contrasts may hinder higher level language development such as vocabulary and reading comprehension.

The results of the speech categorization tasks revealed that the children with poor reading comprehension exhibited the shallower categorization curve than those with typical development. Although both groups exhibited the basic “S” curve shape with similar phonetic boundaries (i.e., crossover between the fourth and fifth sound stimuli of the /pa/–/ta/ continuum), the RD group showed a higher identification rate of stimuli in a single category than did the control group. Within-category contrasts were highly differentiated in the RD group than the control group, consistent with the findings of Serniclaes et al. (2001). This result suggested that the within-category items may be perceived as allophones of the same phoneme. The less consistent labeling of endpoint tokens and consequential shallower identification function implies less categorization in phonetic representations. This lower categorical perception is similar to observations in studies of children with language impairment (Sussman, 1993; Ziegler et al., 2005; Robertson et al., 2009). Notably, this finding supports those of previous studies (Joanisse et al., 2000; Robertson et al., 2009), in which dyslexic children without concomitant language impairment exhibited seemingly average categorization abilities. In the present study, the slope of identification curve was correlated with vocabulary and reading comprehension performance but not with word recognition. This finding suggested that categorical speech deficits are more closely linked to language processing deficits than word decoding problems.

Examinations of the profile of categorical perception in the children with poor reading comprehension revealed that such children exhibit a higher proportion of within-category discrimination but a lower accuracy of the between-category discrimination condition than do those in the control group. Moreover, the higher discrimination proportions of within-category pairs are associated with individuals’ shallower identification curve, which suggests the presence of less abrupt phonetic boundaries in children with poor reading comprehension. The difficulties of the children in discriminating and categorizing speech sounds may result in the poor performance on identifying or degrading the representations of phonemes, the deficiencies of phonological representations of words in children’s mental lexicon, and the problems in spelling-to-sound learning.

In the present study, significant correlations were observed between speech perception measures (e.g., lexical tone discrimination, consonant discrimination, and categorical speech perception) and measures of language and reading comprehension. Specifically, stronger correlations were observed between speech perception measures and language and reading comprehension performance than between speech perception measures and word recognition scores. In line with the simple view of reading, for which linguistic comprehension and word decoding skills are core components of reading comprehension (Gough and Tunmer, 1986), our results indicate that speech perception is essential for linguistic comprehension and, as a result, contributes to reading comprehension.

Among the speech perception measures, the sensitivity to distinguish lexical tones is more strongly correlated with language and reading comprehension than consonant discrimination. Because lexical tones in Mandarin differentiate lexical meanings between syllables and characters, poorer lexical tone perception ability may lead to the inadequate development of mental representations of tonal categories (Zhang et al., 2012), which in turn may result in poor vocabulary knowledge and comprehension. Thus, deficits in lexical tone perception contributes to language and reading comprehension difficulties in speakers of tonal languages such as Mandarin (Chen and Liu, 2010; Liu et al., 2013). Furthermore, controlling for age and non-verbal IQ, the hierarchical multiple regression analysis results suggested that lexical tone perception ability accounts for the variances of the word recognition, language and reading comprehension. Results of the current study indicate that children experiencing reading comprehension difficulties exhibit general speech perception deficits at both the segmental and suprasegmental levels. It remains to be seen whether future research would bear out such a prediction in languages with alphabetic writing systems (e.g., English).

### Limitations

Notably, children with poor reading comprehension have heterogeneous profiles of oral language, word decoding, and reading comprehension. As opposed to word decoding performance, we used reading comprehension performance as the criterion to identify children with poor reading difficulty. In the present study, the children with poor reading comprehension could have exhibited concomitant oral language and word reading deficiencies. Therefore, a particular regression model was used to control for age, non-verbal IQ, vocabulary comprehension, and word recognition and confirmed that sensitivity to tone discrimination in Mandarin-speaking children is the contributor to reading comprehension development. This regression model provides further evidence that speech perception deficits, particularly those related to lexical tone perception, contribute to reading comprehension development in Mandarin-speaking school-aged children.

There were some methodological issues that need to be taken into consideration in relation to the findings of this study. First, the current study included a modest sample size that could limit the generalizability of the findings to the population. Furthermore, this sample did not allow for classification of subgroups of children with reading comprehension difficulties. Additionally, the use of a single measure and a cutoff score prescribed by that measure may raise concerns related to possible false negative or false positive classification of children with reading comprehension difficulties. Future studies are needed that recruit larger samples and use multiple measures to classify children first for reading comprehension difficulty and then into subgroups based on their word decoding skills and linguistic comprehension skills (i.e., poor reading skills, poor comprehension; Cain, 2016). Extending this study in this way would further clarify the effects of speech perception deficits on word decoding or reading comprehension deficiencies.

Although significant concurrent and predictive relations were observed between speech perception, language comprehension, word decoding and reading comprehension, these correlations could not be reliably interpreted in a directional way. Whether poor reading comprehension is a consequence of basic-level perceptual deficits, such as those in the discrimination and categorical perception of speech sounds, or is mediated by other phonological processes, such as phonological awareness and working memory, remains unknown. Previous studies (e.g., Robertson et al., 2009; Hakvoort et al., 2016) have reached a consensus that phonological processing deficits exert a critical effect on word decoding difficulties, but speech perception deficits may not be directly or significantly associated with dyslexia. Moreover, the relationship between speech perception deficits and phonological processing may be highly complex. Future studies should consider speech perception and phonological processes when analyzing poor reading comprehension in children.

## Conclusion

The present study revealed that Mandarin-speaking children with poor reading comprehension have impaired speech discrimination and categorical perception relative to their age-matched peers. Speech perception abilities are linked to children’s language and reading comprehension.

## Author Contributions

H-ML developed the study concept. Both H-ML and F-MT contributed to the study design, data collection, and data analysis. H-ML drafted the manuscript, and F-MT provided critical revisions. Both authors approved the final version of the manuscript for submission.

## Conflict of Interest Statement

The authors declare that the research was conducted in the absence of any commercial or financial relationships that could be construed as a potential conflict of interest.
